# 瘦素/ERK信号在云锡矿粉诱导大鼠II型肺泡上皮细胞转化中的作用

**DOI:** 10.3779/j.issn.1009-3419.2023.102.37

**Published:** 2023-10-20

**Authors:** Xiong HU, Cong YAN, Yu ZHANG, Guiyun LI, Zheyan ZHOU, Yonghua RUAN, Shiyue LIU, Li BIAN

**Affiliations:** ^1^650031 昆明，昆明医科大学第一附属医院病理科; ^1^Department of Pathology, The First Affliated Hospital of Kunming Medical University, Kunming 650031, China; ^2^650500 昆明，昆明医科大学基础医学院; ^2^Basic Medicine of Kunming Medical University School of Basic Medicine, Kunming 650500, China; ^3^650011 昆明，云南省第三人民医院病理科; ^3^Department of Pathology, The Third People’s Hospital of Yunnan Province, Kunming 650011, China

**Keywords:** 瘦素, 肺泡II型上皮细胞, 转化, ERK信号通路, Leptin, Alveolar type II epithelial cells, Transformation, ERK signaling pathway

## Abstract

**背景与目的:**

目前，云南个旧锡矿有大量矿工从事开采工作，这种职业环境与接触粉尘颗粒、重金属、多环芳烃和放射性氡有关，大大增加了患肺癌的风险。本研究旨在探讨在云锡矿粉诱导大鼠肺泡II型上皮细胞（immortalized rat alveolar cells type II, RLE-6TN）恶性转化过程中，瘦素（leptin）及其介导的细胞外调节蛋白激酶（extracellular regulated protein kinase, ERK）信号通路所起的作用。

**方法:**

采用200 μg/mL的云锡矿粉隔代毒染RLE-6TN至第9代，建立毒染细胞模型，命名为R_200_细胞，正常培养组命名为R细胞，通过Western blot法检测两种细胞leptin受体的表达情况。通过MTT法筛选出leptin及丝裂原活化蛋白激酶激酶（mitogen-activated protein kinase kinase, MEK）抑制剂（U0126）对R_200_细胞的最佳作用浓度。自第20代起，将R组、R_200_组细胞分别与leptin及MEK抑制剂U0126共培养，对各组细胞的形态改变进行观察，并利用苏木素-伊红（hematoxylin-eosin, HE）染色技术鉴别第40代细胞的形态学差异，通过刀豆凝集素A（concanavalin A, ConA）及锚着独立性生长实验法检测细胞恶性转化情况。通过Western blot法检测leptin作用后上皮细胞ERK信号通路的变化。

**结果:**

ng/mL时，其促增殖效应最为显著，30 μmol/L U0126可抑制毒染细胞R_200_增殖，与对照组相比具有统计学差异（P<0.05）。自第25代起，leptin诱导的R_200_组（R_200_L组）细胞形态发生变化，至第30代出现恶性转化，至第40代时恶性转化特征明显；而R_200_组细胞及U0126诱导的R_200_组（R_200_LU组）细胞则在第40代时才出现恶性转化特征。R_200_L组细胞凝集速度较R_200_LU组快，其余各组细胞P30出现凝集，且随ConA浓度增加，细胞凝集速度加快。R_200_L组细胞自P40可见克隆形成，克隆形成率为2.25‰±0.5‰，R_200_LU组及R_200_组未见克隆集落。R_200_L组细胞pERK表达增强；加入U0126阻断后，R_200_L组细胞pERK磷酸化水平降低。

**结论:**

Leptin可以促进云锡矿粉毒染肺上皮细胞的恶性转化，ERK信号通路可能是其促进云锡矿粉引发的肺泡II型上皮细胞转化的重要途径。

## Abstract (en)

**Background and objective:**

Currently, a significant number of miners are involved in mining operations at the Gejiu tin mine in Yunnan. This occupational setting is associated with exposure to dust particles, heavy metals, polycyclic aromatic hydrocarbons, and radioactive radon, thereby significantly elevating the risk of lung cancer. This study aims to investigate the involvement of leptin-mediated extracellular regulated protein kinase (ERK) signaling pathway in the malignant transformation of rat alveolar type II epithelial cells induced by Yunnan tin mine dust.

**Methods:**

Immortalized rat alveolar cells type II (RLE-6TN) cells were infected with Yunnan tin mine dust at a concentration of 200 μg/mL for nine consecutive generations to establish the infected cell model, which was named R_200_ cells. The cells were cultured normally, named as R cells. The expression of leptin receptor in both cell groups was detected using the Western blot method. The optimal concentration of leptin and mitogen-activated protein kinase kinase (MEK) inhibitor (U0126) on R_200_ cells was determined using the MTT method. Starting from the 20^th^ generation, the cells in the R group were co-cultured with leptin, while the cells in the R_200_ group were co-cultured with the MEK inhibitor U0126. The morphological alterations of the cells in each group were visualized utilizing hematoxylin-eosin staining. Additionally, concanavalin A (ConA) was utilized to detect any morphological differences, and an anchorage-independent growth assay was conducted to assess the malignant transformation of the cells. The changes in the ERK signaling pathway in epithelial cells after the action of leptin were detected using the Western blot method.

**Results:**

Both the cells in the R group and R_200_ group express leptin receptor OB-R. Compared to the R_200_ group, the concentration of leptin at 100 ng/mL shows the most significant pro-proliferation effect. The proliferation of R_200_ cells infected with the virus is inhibited by 30 μmol/L U0126, and a statistically significant divergence was seen when compared to the control group (P<0.05). Starting from the 25^th^ generation, the cell morphology of the leptin-induced R_200_ group (R_200_L group) underwent changes, leading to malignant transformation observed at the 30^th^ generation. The characteristics of malignant transformation became evident by the 40^th^ generation in the R_200_L group. In contrast, the other groups showed agglutination of P40 cells, and the speed of cell aggregation increased with an increase in ConA concentration. Notably, the R_200_L group exhibited faster cell aggregation compared to the U0126-induced R_200_ (R_200_LU) group. Additionally, the cells in the R_200_L group were capable of forming clones starting from P30, with a colony formation rate of 2.25‰±0.5‰. However, no clonal colonies were observed in the R_200_LU group and R_200_ group. The expression of phosphorylated extracellular signal-regulated kinase (pERK) was enhanced in cells of the R_200_L group. However, when the cells in the R_200_L group were treated with U0126, a blocking agent, the phosphorylation level of pERK decreased.

**Conclusion:**

Leptin can promote the malignant transformation of lung epithelial cells infected by mine dust, and the ERK signaling pathway may be necessary for the transformation of alveolar type II epithelial cells induced by Yunnan tin mine dust.

云南个旧锡矿工人群体被视为肺癌高发群体之一，鳞癌是其主要的病理组织学类型，且癌灶及癌旁肺组织常伴发弥漫性纤维化^[1]^。课题组既往研究^[2]^发现：云锡矿粉可引发大鼠肺部出现肺纤维组织增生、非典型样增生及癌变，体外实验^[3]^也证实云锡矿粉可诱导人胚肺成纤维细胞WI-38活化，激活的成纤维细胞能够增强BEAS-2B支气管上皮细胞的恶性转化过程，但其可能机制尚需进一步探索。Leptin是由“肥胖基因”（obese gene, Ob gene）编码的一种细胞因子样激素，有研究^[4⇓-6]^证实它在多种恶性肿瘤的发生、发展中发挥着重要作用。课题组前期采用蛋白芯片检测技术发现矿粉诱导活化的成纤维细胞leptin表达明显升高，在本次研究中，我们通过建立leptin、丝裂原活化蛋白激酶激酶（mitogen-activated protein kinase kinase, MEK）特异性抑制剂U0126与矿粉诱导大鼠肺泡II型上皮细胞（immortalized rat alveolar cells type II, RLE-6TN）共培养模型，探索leptin调控矿粉诱导RLE-6TN转化作用及可能机制。

## 1 资料和方法

### 1.1 主要试剂

云锡矿粉（原云锡劳动卫生研究所提供），颗粒粒径中值5.189 μm^[7]^；RLE-6TN细胞株（美国ATCC公司）；DMEM/F12培养液（美国Hyclone）；细胞培养板和培养皿（美国Coring公司）；Recombinant Rat Leptin/OB（rRtLeptin，美国R&D公司）；丝裂原活化蛋白激酶激酶（mitogen-activated protein kinase kinase, MEK）抑制剂U0126、蛋白质定量检测试剂盒（BCA法）（上海碧云天生物技术有限公司）；细胞外调节蛋白激酶（extracellular regulated protein kinase, ERK）1/2、pERK多克隆抗体（美国Proteintech公司）；刀豆凝集素A（concanavalin A, ConA）（美国Sigma公司）；MTT粉剂（Solarbio公司）。

### 1.2 方法

#### 1.2.1 细胞毒染模型的建立

以含10%胎牛血清（fetal bovine serum, FBS）的DMEM/F12完全培养液在37 ^o^C、5% CO_2_、饱和湿度的培养箱中培养RLE-6TN细胞，待细胞处于对数生长期传代，加入含200 μg/mL矿粉的10% DMEM/F12完全培养基进行毒染，隔一代毒染一次，每次处理72 h，至第9代时一共毒染5次。将毒染组细胞命名为R_200_组，相应代次正常培养的RLE-6TN细胞命名为R组。

#### 1.2.2 rRtLeptin及MEK抑制剂U0126对毒染细胞恶性转化的影响

（1）筛选rRtLeptin及MEK抑制剂U0126作用毒染细胞的最适浓度：取对数生长期的R_200_组细胞，制备单细胞悬液，以每孔1×10^3^个细胞的密度接种于96孔板，24 h后更换无血清DMEM/F-12培养基继续培养12 h，分别加入含rRtLeptin浓度为0、50、100、200、500、800 ng/mL的无血清培养基继续培养12 h；取对数生长期的R_200_组细胞，制备单细胞悬液，以每孔1×10^3^个细胞的密度接种于96孔板，24 h后更换无血清DMEM/F-12培养基继续培养12 h，分别加入含U0126浓度10、20、30、40、50 μmol/L的无血清培养基孵育，1 h后更换为完全培养基继续培养12 h；收集上述各组细胞，每孔加入20 μL 5 mg/mL的MTT液和180 μL无血清培养基，培养4 h后，弃去上清液，每孔加入150 μL DMSO，震荡10 min，使用酶标仪在492 nm波长下测定每个孔的吸光度值（OD值）。（2）选用100 ng/mL的rRtLeptin和30 μmol/L的U1026进行后续实验，取处于对数生长期的R_200_组细胞，更换为无血清DMEM/F-12培养基，继续培养12 h，按下列分组添加试剂：R_200_组为对照组，添加PBS溶液，R_200_L组添加100 ng/mL rRtLeptin；R_200_LU组添加100 ng/mL rRtLeptin+30 μmol/L U0126。

上述各组细胞培养箱中继续培养12 h，分别收集培养第25代（P25）、30代（P30）、40代（P40）细胞，利用倒置显微镜对细胞形态学变化进行观察，并行苏木素-伊红（hematoxylin-eosin, HE）染色进行ConA凝集反应和锚着独立生长实验，观察细胞功能差异。

#### 1.2.3 HE染色及ConA凝集实验

HE染色：收集P40 R_200_组和R_200_L两组细胞制备细胞爬片，苏木素染色样品5 min后，使用1%盐酸酒精进行分化处理、温水返蓝，0.5%伊红染色1 min，采取梯度酒精脱水、透明、中性树胶封片，镜下观察。ConA凝集实验：收集P25和P30的各组细胞，制备单细胞悬液并调整至5×10^4^个/mL的细胞密度，加入不同浓度的ConA溶液（0、25、50、100、200 μg/mL）在室温下进行显微镜观察，如果细胞在30 min内出现凝集现象，则判定为阳性。

#### 1.2.4 细胞凝集现象观察实验

通过制备单细胞悬液并调整细胞密度至5×10^4^个/mL，随后加入不同浓度的ConA溶液（0、25、50、100、200 μg/mL）。在室温条件下，通过显微镜观察30 min，如果细胞在30 min内出现凝集现象，则判定为阳性。

#### 1.2.5 锚着独立性生长实验

制备0.6%的底层琼脂注入6孔板中，取P30和P40 R_200_各组细胞，用2×DMEM/F-12培养基（含有2×抗生素和20% FBS）将细胞密度调整至1×10^3^个/mL，与0.7%琼脂糖按1:1比例混匀，注入铺有底层琼脂的6孔培养板。待上层琼脂凝固后，在37 ^o^C、5% CO_2_、饱和湿度孵育箱中，将细胞培养至14 d，每3 d更换一次培养液。利用显微镜观察并记录超过50个细胞的克隆数量并计算克隆形成率。

#### 1.2.6 Western blot

收集细胞用细胞裂解液处理，提取总蛋白。通过BCA试剂盒测定各组细胞的蛋白浓度，调整蛋白浓度为70 μg/18 μL，SDS-PAGE电泳并湿转法转移至PVDF膜，5%牛血清白蛋白室温封闭1 h，1×TBST洗膜；5 mL一抗在4 ^o^C环境下孵育过夜；再使用1×TBST进行3次洗膜；酶标二抗室温下孵育2 h；再使用1×TBST进行3次洗膜；化学发光，显影，定影并采集图像，ImageJ软件对结果进行灰度值测定。

### 1.3 统计学处理

用Graphad Prism 8.0（Graphad software. San Diego, SA）对数据进行分析。以均数±标准差（Mean±SD）的方式来表示所获得的数据。在进行两组数据的比较时采用独立样本t检验作为分析方法；多组数据间比较使用方差分析和LSD-t检验。P<0.05为有统计学差异。

## 2 结果

### 2.1 R组、R_200_组细胞均表达leptin受体OB-R

Western blot检测显示，正常及200 μg/mL矿粉毒染的RLE-6TN中均存在leptin受体（OB-R）蛋白表达，比较两组细胞的OB-R蛋白表达水平，未发现明显差异（图1）。

**图1:**
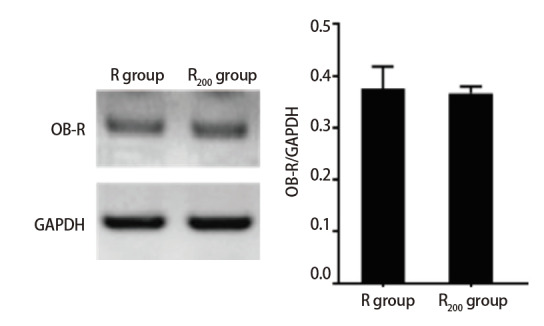
Western blot检测R组、R_200_组细胞leptin受体OB-R表达情况

### 2.2 rRtLeptin和U0126对毒染细胞作用的最适浓度

#### 2.2.1 100 ng/mL rRtLeptin对R_200_组细胞的促增殖效果最佳

用不同浓度leptin处理R_200_组细胞，与对照组（0 ng/mL rRtLeptin）比较，增殖效应明显，且对照组与各组之间OD值差异均有统计学意义（表1，P<0.05）。在0-100 ng/mL之间，rRtLeptin浓度越高，细胞增殖越明显；当rRtLeptin浓度达100 ng/mL时，其促增殖效应最显著；在100-800 ng/mL范围内，rRtLeptin浓度与细胞增殖相关性减弱，细胞增殖趋于稳定，且100 ng/mL作用组与大于100 ng/mL各浓度rRtLeptin作用组相比，差异无统计学意义（P>0.05）；故选100 ng/mL为最适rRtLeptin浓度作用于R_200_细胞（图2A）。

**表1:** 不同浓度的rRtLeptin刺激R_200_细胞后的OD值（Mean±SD）

rRtLeptin	12 h
0 ng/mL	0.2061±0.0053
50 ng/mL	0.1947±0.0025
100 ng/mL	0.2311±0.0036*
200 ng/mL	0.2376±0.0056*
500 ng/mL	0.2241±0.0067*
800 ng/mL	0.2292±0.0084*

*: compared with 0 ng/mL, P<0.05. rRtLeptin: recombinant rat leptin/OB; OD: optical density.

**图2:**
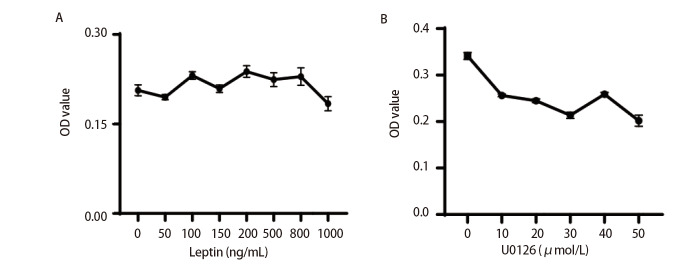
rRtLeptin和U0126作用毒染细胞的浓度筛选折线图

#### 2.2.2 30 μmol/L U0126对R_200_组细胞的生长抑制效果最佳

与对照组相比，10、20、30、40、50 μmol/L的U0126对R_200_细胞生长均有抑制作用（P<0.05）；且在浓度为30和50 μmol/L时，抑制生长效应最明显，但两组之间无统计学差异（表2，P>0.05），因此选浓度为30 μmol/L的U0126用于后续实验（图2B）。

**表2:** 不同浓度的U0126刺激R_200_细胞后的OD值（Mean±SD）

U0126	1 h
0 ng/mL	0.3412±0.0043
10 ng/mL	0.2563±0.0017*
20 ng/mL	0.2449±0.0023*
30 ng/mL	0.2134±0.0039*
40 ng/mL	0.2588±0.0023*
50 ng/mL	0.2019±0.0069*

*: Compared with 0 ng/mL, P<0.05.

### 2.3 rRtLeptin促进矿粉毒染细胞发生恶性转化

#### 2.3.1 rRtLeptin促进矿粉毒染细胞形态改变

用100 ng/mL 的rRtLeptin诱导的R_200_L组细胞至P25时，部分细胞变梭，折光性降低，细胞间的黏附作用减弱，胰酶消化时间明显缩短，单细胞脱落现象显著。至P30时，细胞形态转变为多角且不规则，染色核变大，出现多核和巨核现象，核仁明显，显示出恶性转化的特征，至P40恶性转化特征显著；而R_200_组及R_200_LU组细胞在P40时才出现恶性转化特征（图3）。

**图3:**
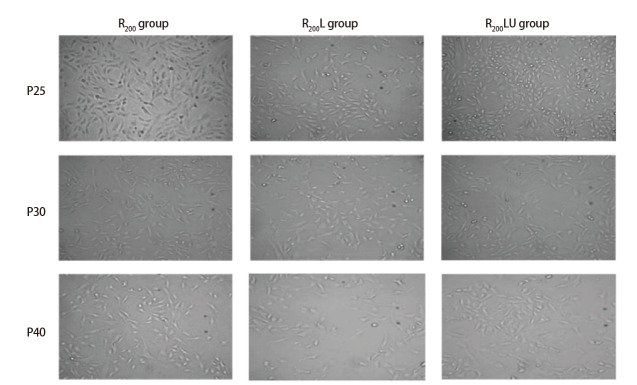
rRtLeptin和U0126对R_200_组细胞形态的影响

将P40的R_200_组和R_200_L组细胞行HE染色发现，R_200_组细胞大小不等、呈短梭形，核畸形、核仁明显肥大、亦可见多个核仁。R_200_L组细胞呈多角不规则形，可见多核、巨核、核仁明显，核分裂像多见，出现明显恶性转化特征（图4）。上述结果表明rRtLeptin可促进云锡矿粉诱导的上皮细胞转化，经MEK阻断剂U0126处理后，其促转化作用减弱。

**图4:**
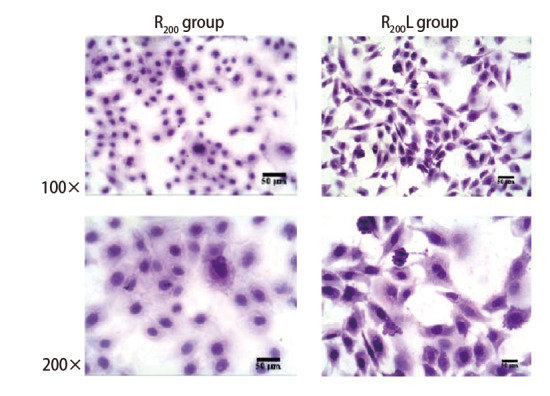
rRtLeptin对R_200_组细胞形态的HE结果图

#### 2.3.2 rRtLeptin增强毒染细胞凝集能力，U0126可减弱其促凝集能力

采用ConA凝集素实验检测P25、P30 R_200_组、R_200_L组、R_200_LU组细胞凝集能力的情况，结果发现，以R组细胞为阴性对照，R_200_L组、R_200_LU组细胞最早在P25、ConA的浓度达到25 μg/mL时出现凝集，凝集时间分别为19、21 min，其余各组在同一Con A浓度下于P30出现凝集，并且随着ConA浓度的不断提升，细胞凝集的速度逐渐加快，凝集的先后顺序为：R_200_L组>R_200_LU组>R_200_组（表3）。表明rRtLeptin可增强毒染细胞的凝集能力，经U0126处理后，其促凝集作用减弱。

**表3:** 第25、30代各组细胞ConA实验结果

ConA	P25 agglutination occurrence time (min)					P30 agglutination occurrence time (min)			
	R group	R_200_ group	R_200_L group	R_200_LU group		R group	R_200_ group	R_200_L group	R_200_LU group
0 μg/mL	-	-	-	-		-	-	-	-
25 μg/mL	-	-	19	21		21	24	18	19
50 μg/mL	-	26	17	18		18	21	16	17
100 μg/mL	-	21	15	15		15	18	13	14
200 μg/mL	-	19	13	14		14	16	12	13

ConA: concanavalin A.

#### 2.3.3 rRtLeptin增强毒染细胞克隆形成能力，U0126可减弱其作用

将P30、P40的R_200_组、R_200_L组、R_200_LU组细胞行锚着独立性生长实验，结果发现，P30各组细胞未出现克隆集落；P40的R_200_L组细胞可见克隆形成，克隆形成率为2.25‰±0.5‰，R_200_组、R_200_LU组及R组细胞均可观察到数个到数十个的细胞聚集成为团状，但并未发现细胞数量超过50的克隆群落（图5）。结果表明rRtLeptin能促进毒染细胞的克隆形成能力，经U0126处理后，其促增殖能力减弱。

**图5:**
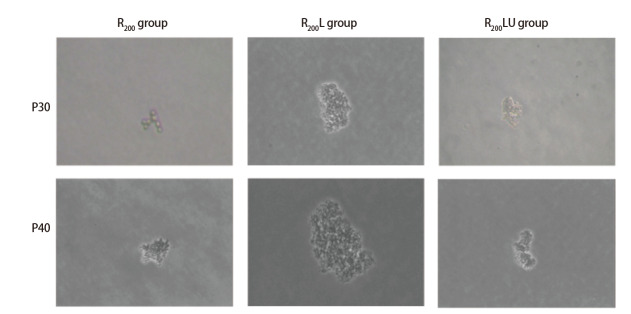
rRtLeptin和U0126作用R_200_组细胞后克隆形成图（×100）

### 2.4 rRtLeptin激活毒染细胞的ERK通路

锚着独立性生长实验及ConA凝集实验结果表明，rRtLeptin可增强第40代R_200_组细胞的克隆形成能力及凝集能力；进一步对该代次的细胞行ERK蛋白及其磷酸化蛋白pERK表达水平检测，结果显示，各组细胞ERK的表达无显著差异（P>0.05）；与R组细胞比较，R_200_组细胞pERK表达增强，经rRtLeptin处理的R_200_L组细胞pERK表达进一步增强，加入U0126阻断后，可部分抑制R_200_L组细胞ERK的磷酸化（图6）。上述结果进一步表明，rRtLeptin可能通过激活ERK信号转导调控毒染细胞的恶性转化及相关功能改变。

**图6:**
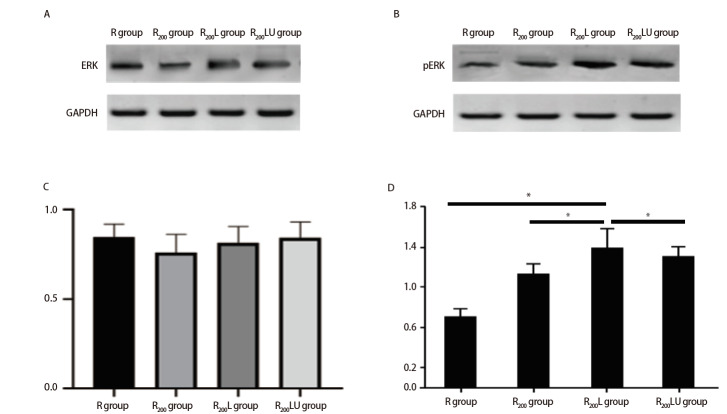
rRtLeptin和U0126作用R_200_组细胞Western blot结果图。A、B：ERK总蛋白的Western blot结果图及其对应的柱状图；C、D：磷酸化ERK蛋白的Western blot结果图及其对应的柱状图。*P<0.05。

## 3 讨论

Leptin是一种具有多种生物学效应的生长因子，主要由脂肪组织合成、分泌，并参与调节脂肪代谢和神经内分泌^[8,9]^，除了存在于能量代谢相关的下丘脑、骨骼肌和脂肪组织，它还普遍存在于全身各组织器官中。近来多项研究结果表明，leptin与多种恶性肿瘤的发生、发展和预后密切相关，其不仅可以促进人乳腺癌细胞、人胰腺癌细胞的体外增殖^[10,11]^，还能降低癌细胞的凋亡能力^[12]^。食管癌、卵巢癌、肺癌、前列腺癌、乳腺癌、大肠癌、胃癌等恶性肿瘤中leptin也呈现高表达^[13⇓⇓⇓⇓⇓-19]^，且血清leptin水平的检测可能提示预后信息。

由于其独特的地域特性，云锡肺癌病理组织学类型主要表现为鳞状细胞癌，且常伴随肺组织广泛的纤维化和肿瘤间质反应^[20]^。肺癌与肺纤维化之间存在密切关系，多数观点认为弥漫性肺纤维化可增加患肺癌的风险，甚至有观点认为肺纤维化可以作为独立的指标来预测肺癌的发生，但其具体的发病机制仍未完全阐明^[21]^。课题组既往研究^[22]^显示正常肺上皮细胞和毒染上皮细胞表达OB-R，经矿粉诱导后的活化成纤维细胞分泌表达的leptin为正常成纤维细胞的17倍。采用rRtLeptin作用于经矿粉诱导的RLE-6TN能够促进其细胞增殖，增强毒染细胞的凝集能力和克隆形成能力等恶性转化能力，且在rRtLeptin浓度为100 ng/mL时细胞增殖效应显著，提示leptin是矿粉诱导成纤维细胞活化促进上皮细胞转化的关键因子。

Leptin通过内分泌、外分泌或旁分泌的方式与其受体相互作用，并通过相应的信号转导途径发挥其多种生物学作用。据文献报道，leptin/OB-R轴可激活ERK通路上调卵巢癌细胞表达基质金属蛋白酶-7（matrix metalloproteinase-7, MMP-7），从而促进卵巢癌的侵袭转移^[23]^，也可诱导肿瘤微环境中巨噬细胞高表达白介素8（interleukin-8, IL-8），为乳腺癌的侵袭迁移提供有利条件^[24]^。在肺癌中，高表达的leptin/leptin受体轴可持续刺激ERK信号通路，促进核因子κB（nuclear factor kappa-B, NF-κB）、即刻早期基因c-fos转录调控基因表达，以促进肿瘤的发生、上皮间质转化及侵袭转移^[16,25]^。Li等^[26]^也发现由肿瘤相关成纤维细胞分泌的leptin可能以旁分泌方式激活丝裂原活化蛋白激酶（mitogen-activated protein kinase, MAPK）/ERK1/2和磷脂酰肌醇3激酶/蛋白激酶B（phosphatidylinositol-3-kinase/protein kinase B, PI3K/AKT）信号通路促进非小细胞肺癌（non-small cell lung cancer, NSCLC）细胞的增殖和迁移。本研究采用ERK信号通路特异性抑制剂U0126作用于矿粉诱导的RLE-6TN，发现U0126可逆转leptin促毒染细胞的增殖、恶性转化、凝集及克隆形成能力，提示ERK信号通路在leptin促进毒染上皮细胞转化过程中发挥关键作用，其作用机制有待进一步研究。在本次研究中，我们发现leptin可能通过ERK信号通路促进矿粉诱导上皮细胞的转化，提示leptin-OB-R/ERK轴作为沟通成纤维细胞活化和肺上皮细胞恶性转化间关系的重要桥梁，可为探讨肿瘤发生发展过程中肿瘤间质与肿瘤细胞的相互交联提供新的研究思路。

**Competing interests**

The authors declare that they have no competing interests.
